# Henagliflozin attenuates adverse ventricular remodeling and improves cardiometabolic parameters in patients with type 2 diabetes and hypertension: real-world evidence from a propensity score-matched analysis

**DOI:** 10.1186/s12933-026-03285-y

**Published:** 2026-07-11

**Authors:** Nianfang Luo, Yutao Liu, Yue Zhang, Bingxi Liu, Mengjiao Li, Kangyan Guo, Liuren Zhang, Jingyi Zhang

**Affiliations:** Department of Cardiology, Jianghan University Affiliated Hubei Third People’s Hospital, Wuhan, 430022 Hubei China

**Keywords:** Type 2 diabetes mellitus, Hypertension, Henagliflozin, Ventricular remodeling

## Abstract

**Background:**

Henagliflozin has demonstrated favorable glycemic and metabolic effects in phase III trials. However, real-world evidence regarding its impact on cardiac structure—particularly left ventricular mass index (LVMI)—in patients with type 2 diabetes mellitus (T2DM) and concomitant hypertension remains lacking.

**Methods:**

In this single-center, retrospective cohort study, 183 patients with T2DM and hypertension were enrolled. After 1:1 propensity score matching, 132 patients (henagliflozin group, *n* = 66; control group, *n* = 66) were included in the primary analysis. The henagliflozin group received henagliflozin 10 mg once daily added to standard-of-care therapy, whereas the control group received standard therapy alone. The primary outcome was the change in LVMI from baseline to 6 months. Secondary outcomes included changes in other echocardiographic parameters (LVEF, E/e′, left atrial diameter, LVEDd, IVSd, LVPWT), glycemic indices (FPG, HbA1c), blood pressure, body mass index (BMI), inflammatory markers, and renal function.

**Results:**

At 6 months, LVMI decreased in the henagliflozin group (from 87.90 ± 18.32 to 85.28 ± 18.20 g/m^2^; mean change − 2.62 ± 13.00 g/m^2^) but increased in the control group (from 83.46 ± 18.22 to 87.81 ± 16.53 g/m^2^; mean change + 4.35 ± 15.88 g/m^2^; between-group *P* = 0.007). Additionally, henagliflozin treatment was associated with significant reductions in left atrial diameter (between-group *P* = 0.035), E/e′ ratio (*P* = 0.044), interventricular septal thickness (*P* = 0.030), and left ventricular posterior wall thickness (*P* = 0.018), as well as a modest increase in LVEF (*P* = 0.045). Compared with the control group, the henagliflozin group also exhibited greater reductions in fasting plasma glucose (− 0.97 vs. + 0.16 mmol/L; *P* = 0.037), HbA1c (− 0.42% vs. + 0.04%; *P* = 0.021), systolic blood pressure (− 9.45 vs. − 1.95 mmHg; *P* = 0.023), diastolic blood pressure (− 3.38 vs. 0.68 mmHg; *P* = 0.029), and BMI (− 0.45 vs. 0.00 kg/m^2^; *P* = 0.023).

**Conclusions:**

In patients with T2DM and hypertension, the addition of henagliflozin to standard therapy over 6 months was associated with significant attenuation of adverse ventricular remodeling—as evidenced by reductions in LVMI, left atrial diameter, and diastolic filling indices—alongside clinically meaningful improvements in glycemic control, blood pressure, and body weight.

**Graphical abstract:**

**Figure:**
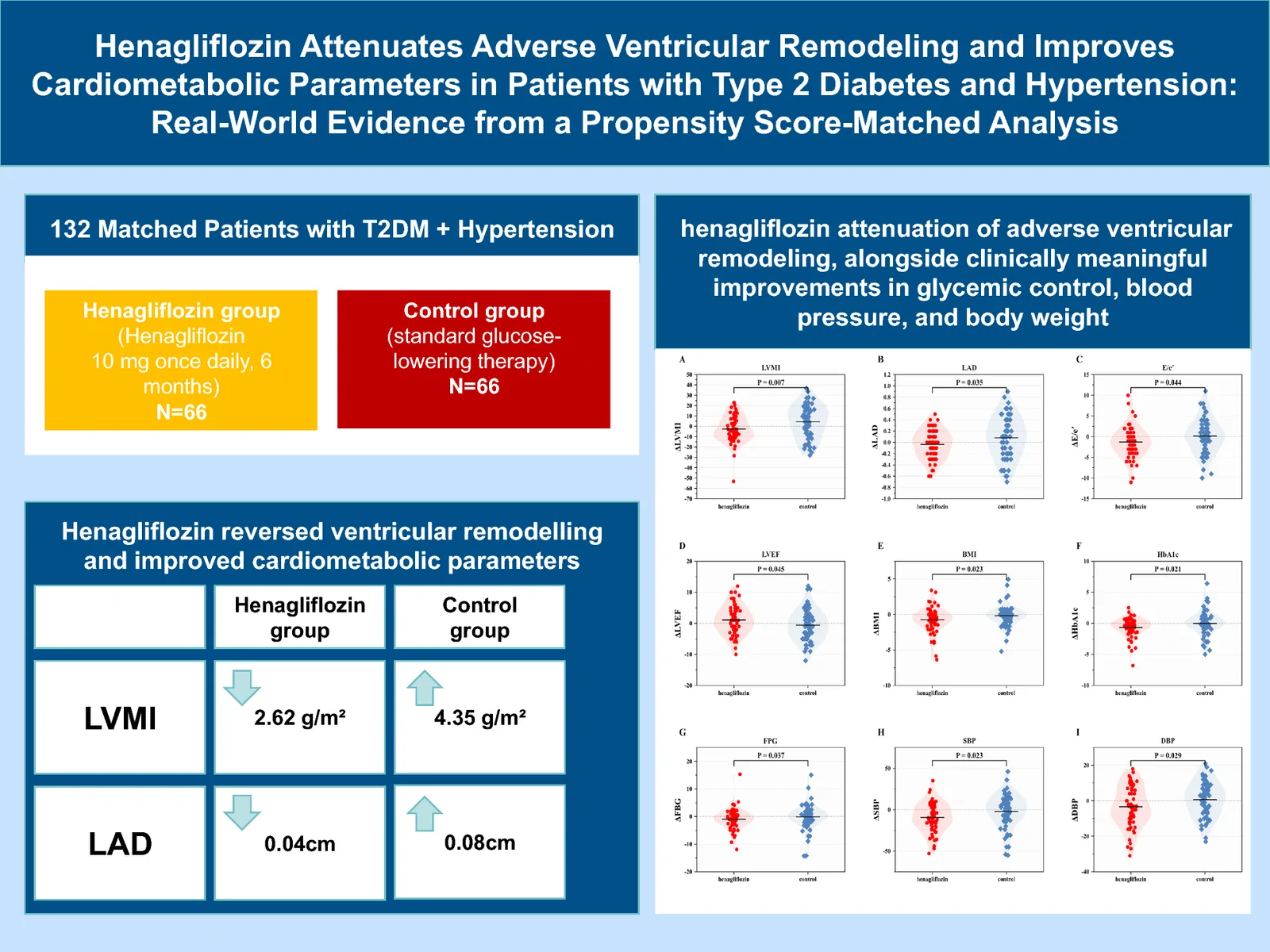

**Supplementary Information:**

The online version contains supplementary material available at https://doi.org/10.1186/s12933-026-03285-y.

## Introduction

Type 2 diabetes mellitus (T2DM) and hypertension frequently coexist and synergistically drive adverse cardiovascular outcomes. T2DM affects approximately 537 million individuals globally (140 million in China, projected to exceed 174 million by 2045) [1]; hypertension affects over 1.2 billion adults worldwide with a prevalence exceeding 45% in Chinese adults [2]. Up to 73.6% of T2DM patients have comorbid hypertension [3], which together more than doubles the risks of cardiovascular death and all-cause mortality compared with either condition alone [4]; yet fewer than 7% of patients achieve simultaneous control of all cardiovascular risk factors.

SGLT2 inhibitors (SGLT2i) have transformed the management of this population. Landmark trials EMPA—REG OUTCOME for cardiovascular outcomes [5], DAPA-HF and DELIVER for heart failure regardless of ejection fraction [6, 7], and DAPA-CKD for renal protection [8] have led major international and Chinese guidelines to recommend SGLT2i as cornerstone therapy for patients with T2DM and comorbid cardiovascular or renal disease [9]. Henagliflozin, the first domestically developed SGLT2i in China [10], demonstrated significant HbA1c reductions (approximately 0.76–0.90%) with body weight and blood pressure benefits and a favorable safety profile in Phase III trials [11]; a trial in dialysis patients with heart failure is currently underway [12]. However, existing evidence is limited to short-term glycemic endpoints (24–52 weeks) in selected populations, leaving long-term cardiovascular outcomes, renal trajectories, and cardiac structural effects uncharacterized in real-world settings [13, 14].

Critically, no real-world data exist on henagliflozin’s impact on cardiac structure particularly left ventricular mass index (LVMI) in patients with T2DM and hypertension. This study provides the first propensity score-matched real-world evaluation of henagliflozin’s effects on LVMI, other echocardiographic indices, glycemic control, blood pressure, body weight, and safety in this high-risk population.

## Methods

### Study design and participants

This was a single-center, retrospective cohort study conducted at the Third People’s Hospital of Hubei Province. Consecutive patients with a confirmed diagnosis of T2DM complicated by hypertension who were hospitalized or attended outpatient follow-up between January 2024 and January 2025 were screened for eligibility. The study protocol was approved by the Ethics Committee of the Third People’s Hospital of Hubei Province (No. KY2025022). The requirement for written informed consent was waived owing to the retrospective nature of the study and the use of de-identified data.

Patients were divided into two groups based on their prescribed glucose-lowering regimen: the henagliflozin group and the control group. The henagliflozin group comprised patients who received henagliflozin 10 mg once daily in addition to standard glucose-lowering therapy for a minimum duration of 6 months. The control group consisted of patients who received standard glucose-lowering therapy without any SGLT2 inhibitor during the study period.

Inclusion criteria were as follows: (1) age between 18 and 100 years; (2) diagnosis of hypertension according to World Health Organization (WHO) criteria; (3) diagnosis of T2DM according to WHO criteria; (4) availability of complete baseline clinical data (collected within 1 month prior to treatment initiation) and follow-up data (collected at ≥ 6 months after treatment initiation). Exclusion criteria included: (1) Non-type 2 diabetes mellitus (e.g., type 1 diabetes, gestational diabetes, monogenic diabetes); (2) Actual duration of henagliflozin treatment < 6 months or lack of baseline and 6-month follow-up examination records; (3) Severe cardiovascular diseases, including secondary hypertension, cardiomyopathy (hypertrophic, restrictive, or dilated), severe valvular heart disease, pericardial disease, or congenital heart disease; (4) History of acute coronary syndrome, myocardial infarction, stroke, severe heart failure, or cardiac surgery within 6 months; (5) Contraindications to henagliflozin or insufficient washout period, including severe allergy, Child–Pugh class C hepatic impairment, severe renal insufficiency (estimated glomerular filtration rate (eGFR) < 30 mL/min/1.73 m^2^), or use of SGLT2 inhibitors within 7 days; (6) Recent acute complications (diabetic ketoacidosis or hyperosmolar hyperglycemic state within 30 days; severe hypoglycemia or diabetic coma within 3 months) or active infection.

Both groups received standard-of-care therapy consisting of conventional glucose-lowering agents (including, but not limited to, metformin, sulfonylureas, dipeptidyl peptidase-4 inhibitors, glucagon-like peptide-1 receptor agonists, and/or insulin), antihypertensive agents, lipid-lowering therapy (statins), and antiplatelet therapy as clinically indicated. A detailed list of concomitant medications is provided in Table 1. Patients in the henagliflozin group additionally received henagliflozin proline tablets (Jiangsu Hengrui Pharmaceuticals Co., Ltd., China) at a dose of 10 mg once daily.

**Table 1:** Baseline characteristics of the study population

	Before PSM (*N* = 183)		*P* value	After PSM (*N* = 132)		*P* value
	Control group (*N* = 99)	Henagliflozin group (*N* = 84)		Control group (*N* = 66)	Henagliflozin group (*N* = 66)	
*Characteristic*						
Age, years	70.48 ± 10.98	66.01 ± 11.04	0.007	68.35 ± 10.92	68.71 ± 9.83	0.841
Gender, n (%)			0.082			0.722
Female	48 (48.48)	30 (35.71)		25 (37.88)	27 (40.91)	
Male	51 (51.52)	54 (64.29)		41 (62.12)	39 (59.09)	
SBP(mmHg)	139.42 ± 17.96	142.40 ± 20.94	0.301	140.44 ± 16.86	142.76 ± 18.60	0.454
DBP(mmHg)	79.00 (73.00, 87.00)	82.00 (72.00, 92.00)	0.275	80.08 ± 9.22	80.55 ± 11.34	0.795
Pulse rate(beats/min)	76.00 (69.00, 84.00)	79.50 (70.75, 88.00)	0.129	77.50 (71.00, 85.75)	76.00 (70.00, 86.75)	0.694
BMI(kg/m^2)^	24.14 (22.14, 25.96)	24.66 (23.03, 27.43)	0.102	24.72 ± 2.76	25.12 ± 3.82	0.493
FPG(mmol/L)	7.20 (6.25, 9.61)	8.52 (6.87, 12.17)	0.009	7.56 (6.40, 11.42)	8.15 (6.49, 10.12)	0.602
CRP(mg/L)	4.66 (1.80, 10.81)	4.50 (1.87, 11.85)	0.798	5.90 (2.19, 12.37)	4.74 (1.72, 10.67)	0.287
ALT(U/L)	20.10 (12.60, 27.45)	19.55 (14.47, 29.12)	0.475	18.90 (12.50, 26.95)	19.40 (13.83, 25.40)	0.572
AST(U/L)	20.40 (16.50, 26.10)	19.65 (17.20, 24.75)	0.705	19.55 (15.22, 25.85)	19.00 (16.85, 22.87)	0.964
TC(mmol/L)	3.78 (3.22, 4.61)	4.24 (3.20, 5.14)	0.282	3.73 (3.30, 5.12)	4.17 (3.17, 5.14)	0.971
TGs(mmol/L)	1.34 (0.94, 2.13)	1.69 (1.17, 2.21)	0.111	1.51 (1.11, 2.43)	1.54 (1.10, 2.04)	0.829
HDL-c(mmol/L)	1.08 (0.93, 1.35)	1.02 (0.91, 1.22)	0.116	1.03 (0.90, 1.31)	1.04 (0.91, 1.27)	0.792
LDL-c(mmol/L)	2.06 (1.46, 2.70)	2.32 (1.62, 3.15)	0.143	2.18 (1.57, 2.91)	2.28 (1.59, 3.15)	0.726
HbA1c(%)	7.07 (6.50, 8.70)	8.10 (7.23, 9.51)	< .001	7.63 (6.74, 9.65)	8.03 (7.03, 8.96)	0.518
Smoking, n (%)	35 (35.35)	35 (41.67)	0.381	26 (39.39)	25 (37.88)	0.858
Drinking, n (%)	18 (18.18)	17 (20.24)	0.724	15 (22.73)	11 (16.67)	0.381
*Comorbidities, n (%)*						
Coronary heart disease	47 (47.47)	35 (41.67)	0.431	25 (37.88)	32 (48.48)	0.219
Heart failure	11 (11.11)	11 (13.10)	0.681	5 (7.58)	11 (16.67)	0.110
Cerebral infarction	22 (22.22)	11 (13.10)	0.110	12 (18.18)	11 (16.67)	0.819
Arrhythmia	9 (9.09)	7 (8.33)	0.857	6 (9.09)	7 (10.61)	0.770
Valvular heart disease	2 (2.02)	1 (1.19)	1.000	2 (3.03)	1 (1.52)	1.000
Malignancy	4 (4.04)	2 (2.38)	0.832	2 (3.03)	1 (1.52)	1.000
Concomitant medications, n (%)						
*Antidiabetic drugs/agents*						
GLP-1 receptor agonist	17 (17.17)	21 (25.00)	0.193	12 (18.18)	17 (25.76)	0.293
Biguanides	29 (29.29)	32 (38.10)	0.208	20 (30.30)	19 (28.79)	0.849
Sulfonylureas	10 (10.10)	5 (5.95)	0.308	7 (10.61)	4 (6.06)	0.345
Glinides	3 (3.03)	1 (1.19)	0.733	2 (3.03)	1 (1.52)	1.000
Thiazolidinediones	11 (11.11)	19 (22.62)	0.036	9 (13.64)	8 (12.12)	0.795
α-glucosidase inhibitors	58 (58.59)	45 (53.57)	0.496	36 (54.55)	37 (56.06)	0.861
DPP-4 inhibitors	22 (22.22)	25 (29.76)	0.245	16 (24.24)	20 (30.30)	0.434
Insulin	34 (34.34)	34 (40.48)	0.392	26 (39.39)	26 (39.39)	1.000
Antihypertensive drugs/agents						
Diuretics	25 (25.25)	16 (19.05)	0.316	16 (24.24)	15 (22.73)	0.837
Calcium channel blockers	54 (54.55)	52 (61.90)	0.315	39 (59.09)	41 (62.12)	0.722
β-blockers	34 (34.34)	32 (38.10)	0.598	22 (33.33)	24 (36.36)	0.715
ACEI/ARB/ARNI	63 (63.64)	61 (72.62)	0.195	45 (68.18)	46 (69.70)	0.851
Antiplatelet agents	78 (78.79)	58 (69.05)	0.133	48 (72.73)	50 (75.76)	0.691
Statins	78 (78.79)	60 (71.43)	0.249	49 (74.24)	50 (75.76)	0.841

Data are expressed as mean ± standard deviation for normally distributed continuous variables, median (P25, P75) for non-normally distributed continuous variables, and percentages for categorical variables

SBP, Systolic blood pressure; DBP, Diastolic blood pressure; BMI, Body mass index; FPG, Fasting plasma glucose; CRP, C-reactive protein; ALT, Alanine aminotransferase; AST, Aspartate aminotransferase; TC, Total cholesterol; TGs, Triglycerides; HDL-c, High-density lipoprotein cholesterol; LDL-c, Low-density lipoprotein cholesterol; HbA1c, Glycated hemoglobin; ACEI/ARB/ARNI, Angiotensin-converting enzyme inhibitors/Angiotensin II receptor blockers/Angiotensin receptor-neprilysin inhibitor

### Data collection and measurements

Baseline demographic and clinical characteristics were extracted from the hospital information system, including the Electronic Medical Record system, the Laboratory Information System, and the Picture Archiving and Communication System. Collected variables included sex, age, smoking and alcohol consumption history, height, body weight, body mass index (BMI), comorbidities, and concomitant medications.

Blood pressure and heart rate were measured by trained personnel using a regularly calibrated upper-arm electronic sphygmomanometer with the patient in a resting state. The mean of three consecutive measurements was recorded. Laboratory parameters were measured from fasting venous blood samples collected in the morning and analyzed in the central clinical laboratory of the Third People’s Hospital of Hubei Province. Parameters included fasting blood glucose, glycated hemoglobin (HbA1c), C-reactive protein (CRP), alanine aminotransferase (ALT), aspartate aminotransferase (AST), N-terminal pro-B-type natriuretic peptide, serum creatinine, eGFR, and lipid profile. The urine albumin-to-creatinine ratio was also determined.

### Outcomes

The primary outcome was the change in left ventricular mass index (LVMI) from baseline to 6-month follow-up. Secondary outcomes included changes in other echocardiographic parameters (left ventricular ejection fraction [LVEF], left atrial anteroposterior diameter [LAD], ratio of early diastolic transmitral flow velocity to early diastolic mitral annular velocity [E/e′ ratio], left ventricular end-diastolic diameter, interventricular septal thickness at end-diastole [IVSd], and left ventricular posterior wall thickness [LVPWT]), as well as changes in systolic blood pressure (SBP) and diastolic blood pressure (DBP), heart rate, glycemic control indices (fasting blood glucose, HbA1c), renal function (serum creatinine, eGFR, urine albumin-to-creatinine ratio), and inflammatory biomarker (CRP).

### Echocardiographic assessment

Transthoracic echocardiography was performed in all enrolled patients at baseline and at 6 months after treatment initiation, using a Philips EPIQ 7 color Doppler echocardiography system (Philips [China] Medical Suzhou Co., Ltd.). All acquisitions and measurements were performed by experienced cardiac sonographers and subsequently reviewed and verified by a senior echocardiographer (associate chief physician or above) who was blinded to the patients’ treatment allocation and clinical data.

### Statistical analysis

Continuous variables were tested for normality using the Shapiro–Wilk test. Normally distributed data are presented as mean ± standard deviation, and non-normally distributed data as median (interquartile range [IQR], Q1–Q3). Categorical variables are summarized as frequencies (percentages).

Within-group comparisons of pre- and post-treatment variables were performed using the paired t-test or the Wilcoxon signed-rank test, as appropriate. Between-group comparisons were conducted using the independent samples t-test or the Mann–Whitney U test for continuous variables, and the chi-square (χ^2^) test for categorical variables.

Because this was a non-randomized retrospective study, we implemented 1:1 propensity score matching (PSM) to minimize potential confounding by indication and to balance baseline characteristics between the henagliflozin and control groups. The propensity score was estimated using a logistic regression model that included the following covariates: age, sex, BMI, fasting plasma glucose (FPG), HbA1c, comorbidities, and concomitant medications, among a total of 21 variables. Matching was performed using a caliper width of 0.02 standard deviations of the logit of the propensity score without replacement. Sensitivity analysis was conducted using the pre-PSM population.

All statistical tests were two-sided, and a *P* value < 0.05 was considered statistically significant. Statistical analyses were performed using SPSS version 27.0 (IBM Corp., Armonk, NY, USA) and R version 4.3.3 (R Foundation for Statistical Computing, Vienna, Austria).

## Results

### Study population demographics

**Fig. 1:**
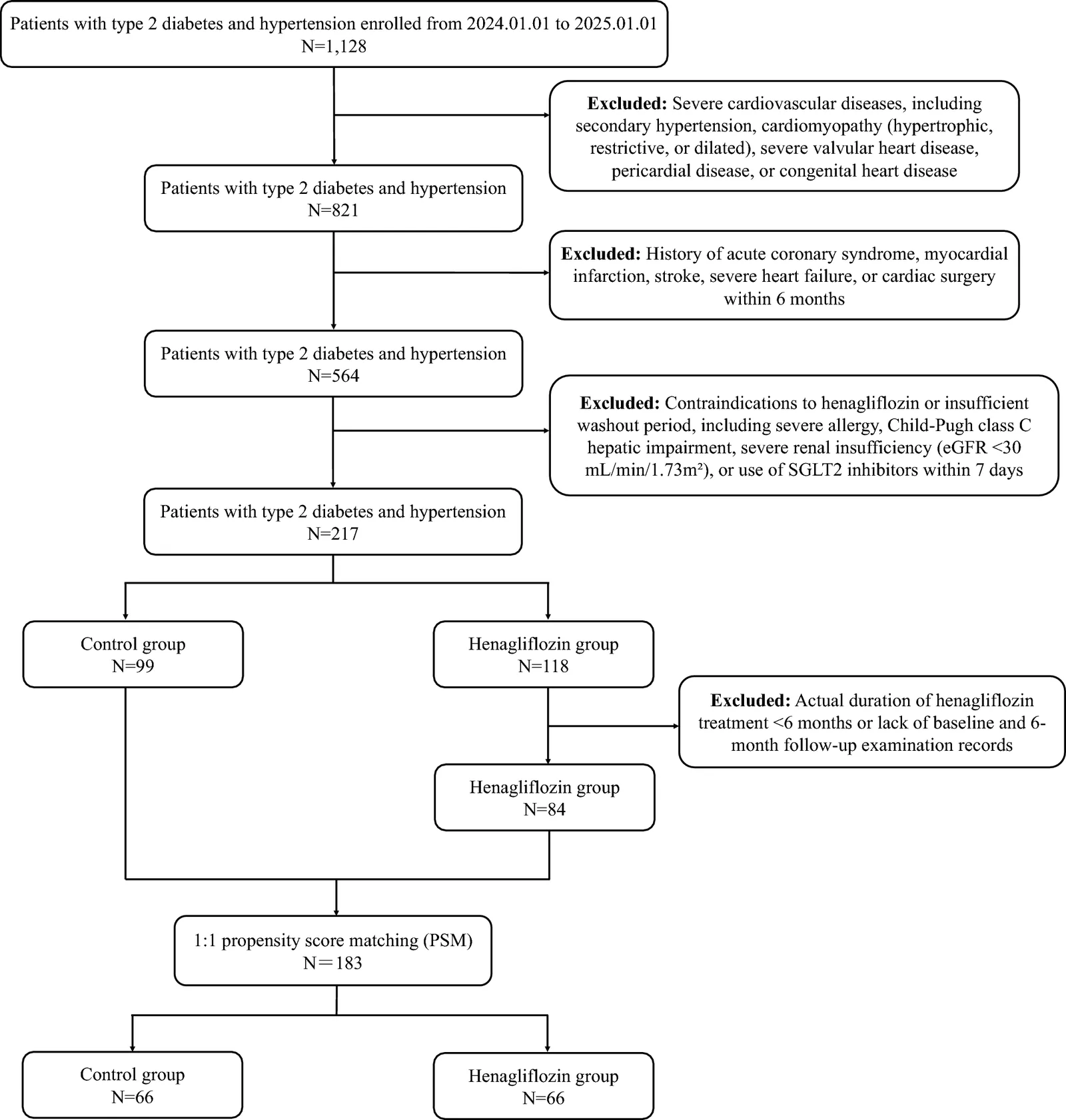
Flow chart

A total of 183 patients with T2DM complicated by hypertension were identified from the institutional database, including 84 who received henagliflozin in addition to standard glucose-lowering therapy and 99 who received standard therapy alone. After 1:1 propensity score matching (caliper width, 0.2 standard deviations of the logit of the propensity score, without replacement), 66 patients were retained in each group. The patient selection process is illustrated in Fig. 1.

Table 1 summarizes the baseline characteristics of the two cohorts. Before PSM, patients in the control group were older and exhibited more favorable short- and long-term glycemic profiles (FPG: 7.20 [6.25, 9.61] vs. 8.52 [6.87, 12.17] mmol/L, *P* = 0.009; HbA1c: 7.07 [6.50, 8.70] vs. 8.10 [7.23, 9.51]%, *P* < 0.001) and were less likely to receive thiazolidinediones (11 [11.1%] vs. 19 [22.6%], *P* = 0.036) compared with henagliflozin users. After PSM, all baseline demographic, clinical, laboratory, and echocardiographic variables were well balanced between the two groups (all standardized differences < 0.1; Table 1), confirming the adequacy of the matching procedure.

### Primary outcome

**Fig. 2:**
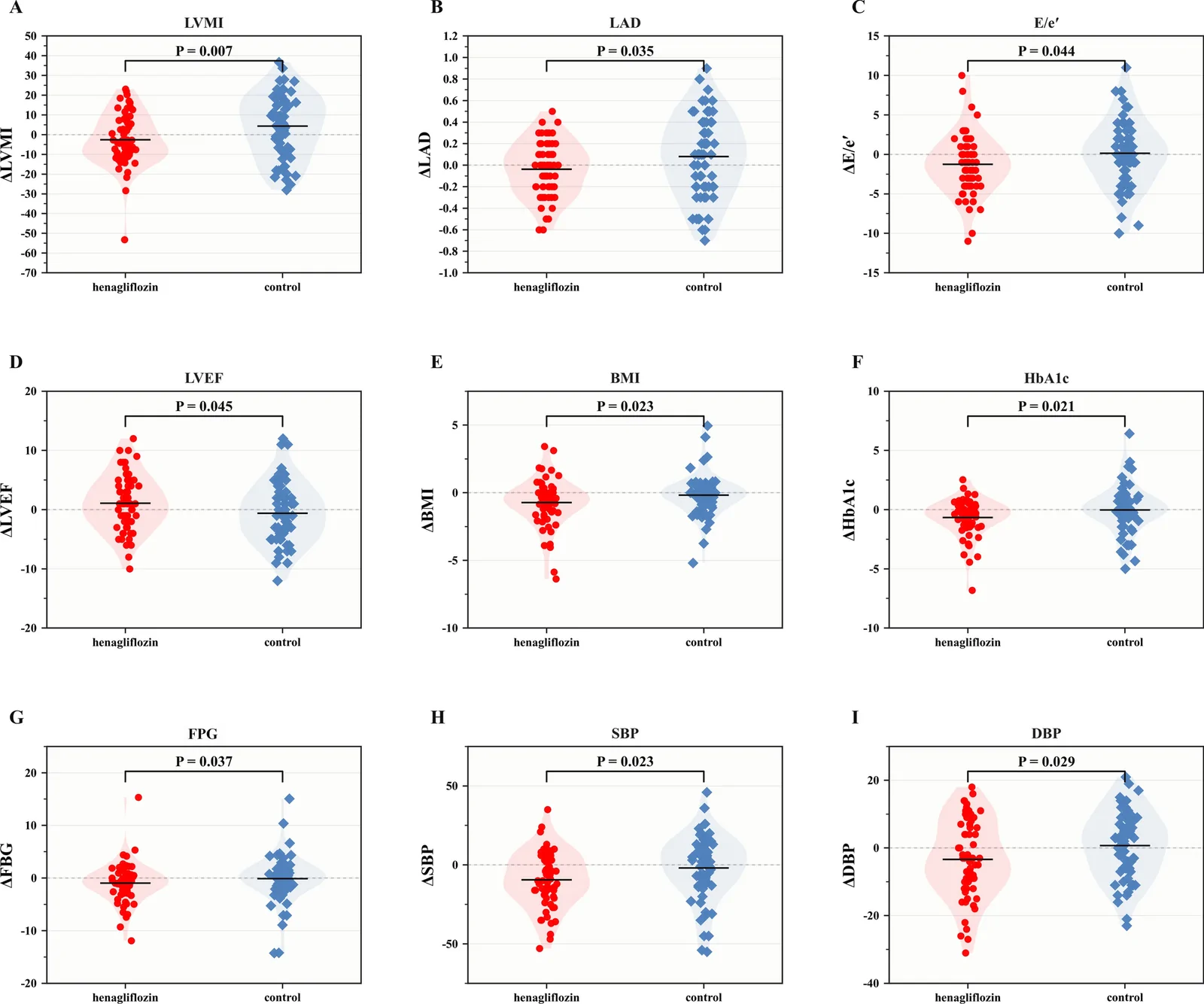
Comparison of changes in cardiac structure, function, and metabolic parameters between the henagliflozin group and the control group after 6 months of treatment. Notes: LVMI, left ventricular mass index (g/m^2^); LAD, left atrial anteroposterior diameter (cm); LVEF, left ventricular ejection fraction (%); BMI, body mass index (kg/m^2^); HbA1c, hemoglobin A1c (%); FPG, fasting plasma glucose (mmol/L); SBP, systolic blood pressure (mmHg); DBP, diastolic plasma pressure (mmHg). All values represent changes from baseline after 6 months of treatment

After 6 months of treatment, LVMI changed divergently between the two groups. In the henagliflozin group, LVMI decreased from 87.90 ± 18.32 g/m^2^ at baseline to 85.28 ± 18.20 g/m^2^ at 6 months (mean change, − 2.62 ± 13.00 g/m^2^), whereas in the control group, LVMI increased from 83.46 ± 18.22 g/m^2^ to 87.81 ± 16.53 g/m^2^ (mean change, + 4.35 ± 15.88 g/m^2^). The between-group difference in LVMI change was statistically significant, with a net difference of − 6.97 g/m^2^ in favor of henagliflozin (*P* = 0.007; Fig. 2).

### Secondary outcome

#### Cardiac structural and functional parameters

Several additional echocardiographic indices of cardiac remodeling showed statistically significant between-group differences favoring henagliflozin (detailed values are presented in Supplementary Table S1). Interventricular septal thickness at end-diastole (IVSd) decreased in the henagliflozin group (median change, 0.00 [− 0.10, 0.07] cm) but increased in the control group (median change, 0.00 [0.00, 0.10] cm; *P* = 0.030). LVPWT showed a similar pattern (henagliflozin: median change, 0.00 [− 0.10, 0.00] cm; control: median change, 0.00 [0.00, 0.10] cm; *P* = 0.018). LAD decreased slightly in the henagliflozin group (mean change, − 0.04 ± 0.25 cm) but increased in the control group (mean change, + 0.08 ± 0.37 cm; *P* = 0.035). LVEDD did not differ significantly between groups (henagliflozin: − 0.04 ± 0.32 cm; control: + 0.07 ± 0.37 cm; *P* = 0.069). LVEF was relatively preserved at baseline in both groups (control: median, 66.00% [IQR, 63.25–69.00]; henagliflozin: 66.00% [62.25–68.75]) and showed a modest but significant between-group difference in change at 6 months (henagliflozin: + 1.08 ± 4.56%; control: − 0.61 ± 4.99%; P = 0.045). The ratio of early diastolic transmitral flow velocity to early diastolic mitral annular tissue velocity (E/e’), a marker of left ventricular diastolic function, improved in the henagliflozin group (mean change, − 1.26 ± 3.68) relative to the control group (mean change, + 0.14 ± 4.18; *P* = 0.044). These changes are summarized graphically in Fig. 2.

#### Glycemic and metabolic parameters

At 6 months, the henagliflozin group exhibited significantly greater reductions in FPG (median change: − 0.97 [IQR, − 2.54 to 0.62] vs. + 0.16 [− 1.51 to 1.22] mmol/L; *P* = 0.037), HbA1c (median change: − 0.42 [− 1.31 to 0.30] vs. + 0.04 [− 0.68 to 0.70]%; P = 0.021), and BMI (median change: − 0.45 [− 1.39 to 0.00] vs. 0.00 [− 0.78 to 0.41] kg/m^2^; *P* = 0.023) compared with the control group (Supplementary Table S1).

#### Hemodynamic parameters

Both systolic and diastolic blood pressure decreased significantly more in the henagliflozin group. SBP decreased by − 9.45 ± 17.34 mmHg in the henagliflozin group versus − 1.95 ± 19.89 mmHg in the control group (*P* = 0.023). DBP decreased by − 3.38 ± 11.29 mmHg in the henagliflozin group, whereas the control group showed a small increase of + 0.68 ± 9.79 mmHg (*P* = 0.029).

#### Inflammatory, hepatic, and renal parameters

No statistically significant between-group differences were observed in the changes from baseline to 6 months for CRP (*P* = 0.881), ALT (P = 0.783), AST (*P* = 0.797), eGFR (*P* = 0.548), serum creatinine (*P* = 0.251), or UACR (*P* = 0.514) (Supplementary Table S1). These findings indicate that, within the 6-month observation period, henagliflozin did not adversely affect hepatic or renal function, and the anti-inflammatory trend was directionally consistent with but quantitatively modest relative to previously reported effects of SGLT2 inhibitors.

### Sensitivity analysis

A sensitivity analysis was performed using the full pre-PSM cohort (N = 183; control, n = 99; henagliflozin, n = 84) to assess the robustness of the primary and secondary findings to the composition of the matched sample. The results, presented in full in Supplementary Table S2, were directionally consistent with those observed in the post-PSM cohort. In the pre-PSM analysis, LVMI differed significantly between groups (*P* = 0.011), and significant improvements in the henagliflozin group were also observed for FPG (*P* < 0.001), HbA1c (*P* < 0.001), SBP (*P* = 0.002), and BMI (*P* = 0.005). Trends toward benefit in IVSd (*P* = 0.078) and LVEF (*P* = 0.072) failed to reach nominal significance but were consistent in direction with the matched analysis. The overall consistency between pre-PSM and post-PSM results supports the robustness of the study conclusions and argues against the findings being artifacts of the propensity score matching process.

### Safety profile

During the 6-month observation period, adverse events in the control group included genitourinary tract infection (*n* = 4) and diabetic ketosis (*n* = 1). In the henagliflozin group, adverse events included hypoglycemia (*n* = 1), hypotension (*n* = 2), genitourinary tract infection (*n* = 6), and diabetic ketosis (*n* = 2). The overall incidence of adverse events did not differ significantly between groups (5/66 [7.6%] in the control group vs. 11/66 [16.7%] in the henagliflozin group (*P* = 0.112). All adverse events resolved following appropriate management, including adjustment of glucose-lowering or antihypertensive regimens and antibiotic therapy where indicated; no recurrence was observed during subsequent follow-up.

## Discussion

The principal finding of this study is that the addition of henagliflozin to standard therapy over 6 months was associated with significant attenuation of adverse cardiac remodeling in patients with T2DM and hypertension. LVMI—the primary outcome—decreased in the henagliflozin group while it increased in the control group, yielding a significant between-group difference (*P* = 0.007). To our knowledge, this is the first study to provide real-world evidence that henagliflozin, the first independently developed SGLT2 inhibitor in China, may confer cardiac structural benefits in this high-risk population. Additionally, henagliflozin was associated with favorable changes in IVSd, LVPWT, LAD, and the E/e′ ratio, as well as modest improvements in blood pressure, glycemic control, and BMI.

### Glycemic, hemodynamic, and metabolic effects

The magnitude of FPG, HbA1c, SBP, DBP, and BMI reductions observed in the henagliflozin group is consistent with its established pharmacodynamic profile. Prior clinical trials have demonstrated that henagliflozin monotherapy reduces HbA1c by approximately 0.76–0.80% with concomitant decreases in body weight (approximately − 1.3 kg) and SBP (− 5 mmHg) over 24–52 weeks, [10–14]; a Bayesian network meta-analysis ranked its glucose-lowering efficacy among the highest within the SGLT2 inhibitor class [14]. In our cohort, henagliflozin treatment was associated with significant reductions in SBP (approximately − 9.5 mmHg), DBP (− 3.4 mmHg), and BMI (− 0.45 kg/m^2^), whereas these parameters remained largely unchanged in the control group. These benefits are particularly relevant for patients with concomitant hypertension and obesity—both highly prevalent in the T2DM population.

### Inflammatory, hepatic, and renal parameters

SGLT2 inhibitors are recognized to exert anti-inflammatory, anti-fibrotic, and antioxidant effects beyond glucose lowering [15–17]. The absence of statistically significant renal benefit at 6 months is consistent with the known hemodynamic time course of SGLT2 inhibitor–associated eGFR changes, which typically exhibit an initial dip (reflecting afferent arteriolar vasoconstriction and reduced intraglomerular pressure) followed by a slower rate of long-term eGFR decline—a pattern observed across landmark trials. [9, 18] The 6-month follow-up in our study captures the early hemodynamic phase but is insufficient to evaluate the longer-term nephroprotective trajectory. Furthermore, the relatively preserved baseline renal function in our cohort (mean eGFR > 80 mL/min/1.73 m^2^) limits the detectable magnitude of renoprotection over 6 months. [19] Larger prospective studies with extended follow-up and enrichment for patients with impaired baseline renal function are warranted to determine whether henagliflozin confers renal benefits in this population commensurate with the established SGLT2 inhibitor class effect.

### Effects on ventricular remodeling and cardiac function

The most salient findings pertain to cardiac structure and function. After 6 months, the henagliflozin group demonstrated favorable changes in multiple echocardiographic parameters indicative of reverse ventricular remodeling: reductions in LVMI, IVSd, and LVPWT were significantly greater than in the control group. The increase in LVMI in the control group is consistent with the natural history of progressive ventricular remodeling driven by sustained hemodynamic and metabolic stress in T2DM with hypertension. These findings are concordant with the EMPA-HEART CardioLink-6 trial, which demonstrated that empagliflozin significantly reduced LVMI versus placebo over 6 months in patients with T2DM and coronary artery disease [20, 21], and meta-analytic evidence that SGLT2 inhibitor use is associated with LVMI reduction, particularly in patients with baseline left ventricular hypertrophy [20, 22].

Henagliflozin was also associated with reductions in LAD and E/e′ ratio, whereas both parameters worsened in the control group. Since left atrial size reflects chronic left ventricular filling pressures and the E/e′ ratio is an established noninvasive index of diastolic function, these data collectively suggest that henagliflozin may improve left ventricular diastolic properties—a finding consistent with the broader class effect of SGLT2 inhibitors on atrial remodeling and diastolic function [23–25]. LVEF was preserved at baseline in both groups; over 6 months, LVEF increased modestly in the henagliflozin group and declined slightly in controls (*P* = 0.045). Although the absolute change is small, the directionally consistent findings across multiple cardiac parameters suggest that henagliflozin may attenuate the progression of adverse ventricular remodeling in patients with T2DM and hypertension.

### Safety profile

The overall incidence of adverse events was low and comparable between groups (7.6% vs. 16.7%; Fisher’s exact test, *P* = 0.112). The numerically higher rate of genitourinary infections in the henagliflozin group (6 vs. 4) is consistent with increased urinary glucose excretion—a well-recognized class effect—and underscores the importance of patient education regarding genital hygiene. The two cases of hypotension likely reflect the mild diuretic and blood pressure–lowering effects of henagliflozin, highlighting the need for blood pressure monitoring in patients receiving concurrent antihypertensive therapy. No new safety signals were identified; however, the limited sample size and 6-month follow-up warrant continued post-marketing surveillance.

## Limitations

Several limitations should be acknowledged. First, the single-center, retrospective design, despite propensity score matching, cannot fully eliminate selection bias or the influence of unmeasured confounders. Second, the modest sample size (132 patients after matching) and 6-month follow-up restrict the power to detect small effect sizes and preclude evaluation of hard cardiovascular or renal endpoints. Third, echocardiographic parameters were derived from two-dimensional imaging using geometric assumptions, which are inherently less precise than cardiac magnetic resonance imaging. Fourth, as an observational study, we did not investigate the mechanistic pathways—such as anti-inflammatory, anti-fibrotic, or metabolic effects—that may mediate the observed reverse remodeling. Fifth, our findings are specific to patients with T2DM and concomitant hypertension and cannot be extrapolated to heart failure patients without these comorbidities; whether henagliflozin benefits patients with isolated heart failure remains an open question under investigation. Sixth, the 6-month follow-up is insufficient to detect long-term renal outcomes, and the study was not enriched for patients with chronic kidney disease; future studies should incorporate longer follow-up and include renal function as a pre-specified primary or key secondary endpoint. Finally, the sample size precluded meaningful subgroup analyses that could identify patient subsets most likely to derive cardiac structural benefit.

## Conclusions

In this retrospective cohort study of patients with T2DM and concomitant hypertension, the addition of henagliflozin to standard-of-care therapy over 6 months was associated with significant reductions in LVMI, left atrial diameter, and markers of diastolic dysfunction, alongside modest improvements in blood pressure, body weight and glycemic control. No significant differences were observed in renal function, or inflammatory markers relative to the control group. The safety profile of henagliflozin was consistent with the known characteristics of the SGLT2i class. These findings provide preliminary real-world evidence that henagliflozin may exert favorable effects on cardiac structure in this high-risk population, supporting its potential role in mitigating adverse ventricular remodeling. Prospective, multicenter randomized controlled trials with extended follow-up are warranted to confirm these observations and to determine whether these structural benefits translate into improved long-term cardiovascular outcomes.

## Supplementary Information

Additional file1 (DOCX 197 KB)

## Data Availability

The datasets analyzed during this analyses are available from the corresponding author Professor Jingyi Zhang (email address: zhangyi@hbzshospital.cn) on reasonable scientific request.

## Abbreviations

ALT: Alanine aminotransferase
AST: Aspartate aminotransferase
BMI: Body mass index
CRP: C-reactive protein
DBP: Diastolic blood pressure
E/e′: Ratio of early diastolic transmitral flow velocity to early diastolic mitral annular velocity
eGFR: Estimated glomerular filtration rate
FPG: Fasting plasma glucose
HbA1c: Glycated hemoglobin
HFpEF: Heart failure with preserved ejection fraction
IQR: Interquartile range
IVSd: Interventricular septal thickness (at end-diastole)
LAD: Left atrial anteroposterior diameter
LVEDd: Left ventricular end-diastolic diameter
LVEF: Left ventricular ejection fraction
LVMI: Left ventricular mass index
LVPWT: Left ventricular posterior wall thickness
PSM: Propensity score matching
SBP: Systolic Blood pressure
SGLT2i: Sodium-glucose cotransporter 2 inhibitor
T2DM: Type 2 diabetes mellitus
UACR: Urine albumin‑to‑creatinine ratio
WHO: World health organization
