# Supplementary material for: Henagliflozin attenuates adverse ventricular remodeling and improves cardiometabolic parameters in patients with type 2 diabetes and hypertension: real-world evidence from a propensity score-matched analysis
Source: Cardiovasc Diabetol. 2026 Jul 11;25:297. doi: 10.1186/s12933-026-03285-y (PMC13640101; doi:10.1186/s12933-026-03285-y)
Supplement: Supplementary file 1 [file 12933_2026_3285_MOESM1_ESM.docx]

CONTENTS

[Supplementary Table S1 Effects of a 6-month use of henagliflozin on clinical parameters	2](#_Toc20620)

[Supplementary Table S2 Effects of a 6-month use of henagliflozin on clinical parameters before propensity score matching	4](#_Toc486)

[Supplementary Table S3 Safety profile of a 6-month use of henagliflozin among patients with type 2 diabetes mellitus and hypertension	6](#_Toc18154)

# Supplementary Table S1 Effects of a 6-month use of henagliflozin on clinical parameters

| Clinical parameters | Control group (N = 66) | Henagliflozin group (N = 66) | *P* value |
| --- | --- | --- | --- |
| FPG (mmol/L) |  |  |  |
| Baseline | 7.56 (6.40, 11.42) | 8.15 (6.49, 10.12) | 0.602 |
| Post-treatment | 7.50 (6.10, 11.40) | 7.25 (5.98, 8.76) ^#^ | 0.242 |
| Δ | 0.16 (-1.51, 1.22) | -0.97 (-2.54, 0.62) | 0.037 |
| HbA1c (%) |  |  |  |
| Baseline | 7.63 (6.74, 9.65) | 8.03 (7.03, 8.96) | 0.518 |
| Post-treatment | 7.69 (6.65, 9.12) | 7.25 (6.58, 8.40) ^#^ | 0.269 |
| Δ | 0.04 (-0.68, 0.70) | -0.42 (-1.31, 0.30) | 0.021 |
| SBP (mmHg) |  |  |  |
| Baseline | 138.38 ± 18.55 | 142.91 ± 18.93 | 0.167 |
| Post-treatment | 136.42 ± 17.40 | 133.45 ± 15.92 ^#^ | 0.308 |
| Δ | -1.95 ± 19.89 | -9.45 ± 17.34 | 0.023 |
| DBP (mmHg) |  |  |  |
| Baseline | 80.08 ± 9.22 | 80.55 ± 11.34 | 0.795 |
| Post-treatment | 80.76 ± 10.10 | 77.17 ± 11.49 ^#^ | 0.059 |
| Δ | 0.68 ± 9.79 | -3.38 ± 11.29 | 0.029 |
| BMI (kg/m2) |  |  |  |
| Baseline | 24.72 ± 2.76 | 25.12 ± 3.82 | 0.493 |
| Post-treatment | 24.54 ± 2.63 | 24.39 ± 3.22 ^#^ | 0.764 |
| Δ | 0.00 (-0.78, 0.41) | -0.45 (-1.39, 0.00) | 0.023 |
| CRP (mg/L) |  |  |  |
| Baseline | 5.90 (2.19, 12.37) | 4.74 (1.72, 10.67) | 0.287 |
| Post-treatment | 4.10 (2.00, 8.68) | 2.75 (1.56, 8.55) ^#^ | 0.482 |
| Δ | -0.38 (-5.67, 1.35) | -0.42 (-3.42, 0.82) | 0.881 |
| ALT (U/L) |  |  |  |
| Baseline | 18.90 (12.50, 26.95) | 19.40 (13.83, 25.40) | 0.572 |
| Post-treatment | 18.70 (13.25, 27.25) | 20.75 (14.22, 27.55) | 0.615 |
| Δ | -1.26 ± 14.29 | -0.60 ± 13.09 | 0.783 |
| AST (U/L) |  |  |  |
| Baseline | 19.55 (15.22, 25.85) | 19.00 (16.85, 22.87) | 0.964 |
| Post-treatment | 20.05 (15.80, 25.73) | 18.60 (15.60, 25.58) | 0.594 |
| Δ | -0.99 ± 9.25 | -0.62 ± 7.34 | 0.797 |
| eGFR (mL/min/1.73m^2^) |  |  |  |
| Baseline | 86.76 (62.67, 102.30) | 85.73 (69.03, 96.98) | 0.836 |
| Post-treatment | 76.49 ± 26.60 ^#^ | 80.54 ± 22.76 | 0.348 |
| Δ | -3.43 (-8.75, 4.09) | -1.42 (-7.93, 3.67) | 0.548 |
| Cr (μmol/L) |  |  |  |
| Baseline | 76.55 (56.18, 97.50) | 75.10 (57.05, 84.97) | 0.808 |
| Post-treatment | 92.48 ± 56.72 ^#^ | 79.14 ± 26.20 | 0.086 |
| Δ | 5.05 (-6.36, 13.66) | 1.25 (-5.05, 8.13) | 0.251 |
| UACR (mg/g) |  |  |  |
| Baseline | 89.48 (17.70, 447.57) | 56.92 (14.75, 278.36) | 0.296 |
| Post-treatment | 43.77 (11.70, 367.43) | 27.16 (8.28, 147.76) ^#^ | 0.139 |
| Δ | -3.15 (-49.63, 52.05) | -3.56 (-62.47, 5.07) | 0.514 |
| LVEDD (cm) |  |  |  |
| Baseline | 4.48 ± 0.43 | 4.58 ± 0.38 | 0.159 |
| Post-treatment | 4.55 ± 0.40 | 4.60 ± 0.42 | 0.455 |
| Δ | 0.07 ± 0.37 | -0.04 ± 0.32 | 0.069 |
| IVSd (cm) |  |  |  |
| Baseline | 1.00 (0.90, 1.00) | 1.00 (0.90, 1.00) | 0.795 |
| Post-treatment | 1.00 (0.90, 1.10) | 0.90 (0.90, 1.00) | 0.014 |
| Δ | 0.00 (0.00, 0.10) | 0.00 (-0.10, 0.07) | 0.030 |
| LVPWT (cm) |  |  |  |
| Baseline | 0.90 (0.90, 1.00) | 1.00 (0.90, 1.00) | 0.747 |
| Post-treatment | 1.00 (0.90, 1.00) | 0.90 (0.90, 1.00) ^#^ | 0.041 |
| Δ | 0.00 (0.00, 0.10) | 0.00 (-0.10, 0.00) | 0.018 |
| LVMI (g/m^2^) |  |  |  |
| Baseline | 83.46 ± 18.22 | 87.90 ± 18.32 | 0.165 |
| Post-treatment | 87.81 ± 16.53 ^#^ | 85.28 ± 18.20 | 0.405 |
| Δ | 4.35 ± 15.88 | -2.62 ± 13.00 | 0.007 |
| E/e' |  |  |  |
| Baseline | 12.00 (10.00, 16.00) | 12.50 (10.00, 15.75) | 0.760 |
| Post-treatment | 13.00 (11.00, 15.00) | 12.00 (9.25, 14.00) ^#^ | 0.074 |
| Δ | 0.14 ± 4.18 | -1.26 ± 3.68 | 0.044 |
| LAD (cm) |  |  |  |
| Baseline | 3.61 ± 0.41 | 3.71 ± 0.55 | 0.230 |
| Post-treatment | 3.69 ± 0.40 | 3.67 ± 0.52 | 0.837 |
| Δ | 0.08 ± 0.37 | -0.04 ± 0.25 | 0.035 |
| LVEF(%) |  |  |  |
| Baseline | 66.00 (63.25, 69.00) | 66.00 (62.25, 68.75) | 0.410 |
| Post-treatment | 65.50 (63.00, 68.00) | 67.00 (64.00, 69.75) | 0.192 |
| Δ | -0.61 ± 4.99 | 1.08 ± 4.56 | 0.045 |

Δ = Post-treatment – Baseline. **#** *P*＜0.05 vs. baseline within the same group at 6 months.Data are expressed as mean ± standard deviation for normally distributed continuous variables, median (P25, P75) for non-normally distributed continuous variables, and percentages for categorical variables.

Abbreviations: SBP, Systolic blood pressure; DBP, Diastolic blood pressure; BMI, Body mass index; FPG, Fasting plasma glucose; CRP, C-reactive protein; ALT, Alanine aminotransferase; AST, Aspartate aminotransferase; HbA1c, Glycated hemoglobin; Cr, Serum creatinine; eGFR, Estimated glomerular filtration rate; UACR, Urine albumin‑to‑creatinine ratio; LVEDD, Left ventricular end-diastolic diameter; IVSd, Interventricular septal thickness at end‑diastole; LVPWT, Left ventricular posterior wall thickness; LVMI, Left ventricular mass index; E/e', Ratio of early diastolic transmitral flow velocity (E) to early diastolic mitral annular tissue velocity (e′); LAD, Left atrial anteroposterior diameter; LVEF, Left ventricular ejection fraction.

# Supplementary Table S2 Effects of a 6-month use of henagliflozin on clinical parameters before propensity score matching

| Clinical parameters | Control group (N = 99) | Henagliflozin group (N = 84) | Statistic | *P* value |
| --- | --- | --- | --- | --- |
| FPG (mmol/L) |  |  |  |  |
| Baseline | 7.20 (6.25, 9.61) | 8.52 (6.87, 12.17) | -2.62 | 0.009 |
| Post-treatment | 7.21 (6.05, 9.12) | 7.14 (5.79, 8.64) ^#^ | -0.95 | 0.340 |
| Δ | 0.09 (-1.22, 1.21) | -1.05 (-3.52, 0.44) | -3.69 | <.001 |
| HbA1c (%) |  |  |  |  |
| Baseline | 7.07 (6.50, 8.70) | 8.10 (7.23, 9.51) | -3.35 | <.001 |
| Post-treatment | 7.10 (6.41, 8.56) | 7.17 (6.53, 8.38) ^#^ | -0.07 | 0.944 |
| Δ | 0.04 (-0.42, 0.54) | -0.54 (-1.58, 0.08) | -4.18 | <.001 |
| SBP (mmHg) |  |  |  |  |
| Baseline | 139.42 ± 17.96 | 142.40 ± 20.94 | -1.04 | 0.301 |
| Post-treatment | 135.00 (125.00, 145.50) | 129.50 (122.00, 143.00) ^#^ | -1.89 | 0.059 |
| Δ | -2.33 ± 18.79 | -11.00 ± 19.06 | 3.09 | 0.002 |
| DBP (mmHg) |  |  |  |  |
| Baseline | 79.00 (73.00, 87.00) | 82.00 (72.00, 92.00) | -1.09 | 0.275 |
| Post-treatment | 79.00 (75.00, 87.50) | 77.00 (73.00, 85.25) | -0.71 | 0.477 |
| Δ | 1.00 (-7.00, 7.00) | -3.00 (-10.25, 7.25) | -1.29 | 0.196 |
| BMI (kg/m2) |  |  |  |  |
| Baseline | 24.14 (22.14, 25.96) | 24.66 (23.03, 27.43) | -1.64 | 0.102 |
| Post-treatment | 23.94 (22.49, 26.08) | 24.51 (22.18, 26.31) ^#^ | -0.63 | 0.531 |
| Δ | 0.00 (-0.70, 0.35) | -0.45 (-1.47, 0.00) | -2.80 | 0.005 |
| CRP (mg/L) |  |  |  |  |
| Baseline | 4.66 (1.80, 10.81) | 4.50 (1.87, 11.85) | -0.26 | 0.798 |
| Post-treatment | 3.20 (1.73, 9.18) | 3.05 (1.59, 8.74) ^#^ | -0.30 | 0.766 |
| Δ | 0.00 (-3.81, 1.50) | -0.50 (-3.85, 0.90) | -0.74 | 0.459 |
| ALT (U/L) |  |  |  |  |
| Baseline | 20.10 (12.60, 27.45) | 19.55 (14.47, 29.12) | -0.71 | 0.475 |
| Post-treatment | 18.60 (13.65, 28.65) | 21.10 (14.90, 28.32) | -1.15 | 0.249 |
| Δ | 0.30 (-6.75, 4.85) | 0.25 (-7.25, 6.12) | -0.34 | 0.732 |
| AST (U/L) |  |  |  |  |
| Baseline | 20.40 (16.50, 26.10) | 19.65 (17.20, 24.75) | -0.38 | 0.705 |
| Post-treatment | 20.70 (16.10, 26.55) | 18.65 (15.57, 25.83) | -1.05 | 0.292 |
| Δ | -0.10 (-4.60, 4.25) | -0.95 (-5.03, 3.55) | -0.33 | 0.742 |
| eGFR (mL/min/1.73m^2^) |  |  |  |  |
| Baseline | 80.18 (60.04, 96.86) | 85.73 (70.02, 100.06) | -1.85 | 0.064 |
| Post-treatment | 73.87 ± 25.99 ^#^ | 83.24 ± 23.64 | -2.53 | 0.012 |
| Δ | -3.17 (-9.08, 3.82) | -1.39 (-7.64, 4.21) | -0.82 | 0.414 |
| Cr (μmol/L) |  |  |  |  |
| Baseline | 75.00 (57.20, 98.50) | 75.10 (56.00, 90.05) | -0.42 | 0.672 |
| Post-treatment | 80.00 (58.15, 103.45) ^#^ | 75.67 (60.40, 92.55) | -0.91 | 0.362 |
| Δ | 4.60 (-6.21, 12.82) | 1.25 (-5.25, 8.55) | -1.19 | 0.235 |
| UACR (mg/g) |  |  |  |  |
| Baseline | 108.20 (22.64, 437.85) | 65.49 (14.56, 468.32) | -0.82 | 0.414 |
| Post-treatment | 51.92 (12.63, 245.84) | 28.61 (8.69, 170.96) ^#^ | -1.67 | 0.095 |
| Δ | -5.98 (-56.41, 25.21) | -4.48 (-97.04, 4.92) | -0.84 | 0.403 |
| LVEDD (cm) |  |  |  |  |
| Baseline | 4.50 (4.20, 4.80) | 4.60 (4.30, 4.90) | -1.93 | 0.054 |
| Post-treatment | 4.50 (4.20, 4.70) | 4.50 (4.30, 4.80) | -0.64 | 0.525 |
| Δ | 0.10 (-0.25, 0.30) | 0.00 (-0.20, 0.20) | -1.85 | 0.065 |
| IVSd (cm) |  |  |  |  |
| Baseline | 1.00 (0.90, 1.00) | 1.00 (0.90, 1.00) | -0.38 | 0.701 |
| Post-treatment | 1.00 (0.90, 1.10) | 0.95 (0.90, 1.00) | -2.29 | 0.022 |
| Δ | 0.00 (-0.10, 0.10) | 0.00 (-0.10, 0.02) | -1.76 | 0.078 |
| LVPWT (cm) |  |  |  |  |
| Baseline | 0.90 (0.90, 1.00) | 1.00 (0.90, 1.00) | -0.45 | 0.649 |
| Post-treatment | 1.00 (0.90, 1.00) | 0.90 (0.90, 1.00) ^#^ | -2.08 | 0.037 |
| Δ | 0.00 (0.00, 0.10) | 0.00 (-0.10, 0.00) | -2.40 | 0.017 |
| LVMI (g/m^2^) |  |  |  |  |
| Baseline | 86.16 ± 19.12 | 86.26 ± 17.19 | -0.04 | 0.969 |
| Post-treatment | 88.93 (74.96, 98.21) | 81.54 (70.33, 93.88) | -2.38 | 0.017 |
| Δ | 4.12 (-8.46, 15.08) | -2.85 (-11.04, 5.90) | -2.54 | 0.011 |
| E/e' |  |  |  |  |
| Baseline | 12.00 (10.00, 16.00) | 12.00 (10.00, 15.00) | -0.06 | 0.951 |
| Post-treatment | 12.00 (10.00, 15.00) | 12.00 (9.00, 14.00) ^#^ | -1.82 | 0.069 |
| Δ | 0.00 (-3.00, 3.00) | -1.00 (-3.00, 1.00) | -2.01 | 0.045 |
| LAD (cm) |  |  |  |  |
| Baseline | 3.60 (3.40, 3.90) | 3.60 (3.38, 4.00) | -0.48 | 0.634 |
| Post-treatment | 3.70 (3.40, 4.00) | 3.60 (3.30, 3.90) | -0.61 | 0.541 |
| Δ | 0.05 ± 0.36 | -0.03 ± 0.26 | 1.65 | 0.101 |
| LVEF (%) |  |  |  |  |
| Baseline | 66.00 (63.50, 69.00) | 66.00 (62.00, 68.00) | -1.00 | 0.317 |
| Post-treatment | 66.00 (63.00, 68.50) | 67.00 (64.00, 69.25) ^#^ | -0.81 | 0.417 |
| Δ | -0.16 ± 5.09 | 1.13 ± 4.45 | -1.81 | 0.072 |

Δ = Post-treatment – Baseline. **#** *P*＜0.05 vs. baseline within the same group at 6 months.Data are expressed as mean ± standard deviation for normally distributed continuous variables, median (P25, P75) for non-normally distributed continuous variables, and percentages for categorical variables.

Abbreviations: SBP, Systolic blood pressure; DBP, Diastolic blood pressure; BMI, Body mass index; FPG, Fasting plasma glucose; CRP, C-reactive protein; ALT, Alanine aminotransferase; AST, Aspartate aminotransferase; HbA1c, Glycated hemoglobin; Cr, Serum creatinine; eGFR, Estimated glomerular filtration rate; UACR, Urine albumin‑to‑creatinine ratio; LVEDD, Left ventricular end-diastolic diameter; IVSd, Interventricular septal thickness at end‑diastole; LVPWT, Left ventricular posterior wall thickness; LVMI, Left ventricular mass index; E/e', Ratio of early diastolic transmitral flow velocity (E) to early diastolic mitral annular tissue velocity (e′); LAD, Left atrial anteroposterior diameter; LVEF, Left ventricular ejection fraction.

# Supplementary Table S3 Safety profile of a 6-month use of henagliflozin among patients with type 2 diabetes mellitus and hypertension

| Group | Hypoglycemia | Hypotension | Genitourinary infection | Diabetic ketoacidosis | Overall |
| --- | --- | --- | --- | --- | --- |
| Control group (N = 99) | 0 | 0 | 9 | 1 | 10 |
| Henagliflozin group (N = 84) | 1 | 2 | 8 | 3 | 14 |
| *p* value | 0.459 | 0.355 | 1.000 | 0.335 | 0.112 |
